# Docosahexaenoic and Eicosapentaenoic Acids Prevent Altered-Muc2 Secretion Induced by Palmitic Acid by Alleviating Endoplasmic Reticulum Stress in LS174T Goblet Cells

**DOI:** 10.3390/nu11092179

**Published:** 2019-09-11

**Authors:** Quentin Escoula, Sandrine Bellenger, Michel Narce, Jérôme Bellenger

**Affiliations:** 1 University of Bourgogne Franche-Comté, UFR Sciences de la Vie, de la Terre et de l’Environnement, Lipides Nutrition Cancer UMR U1231, 6 Boulevard Gabriel, F-21000 Dijon, France; escoula@hotmail.fr (Q.E.); sandrine.bellenger@u-bourgogne.fr (S.B.); 2 INSERM, Lipides Nutrition Cancer UMR1231, F-21000 Dijon, France; 3 LipSTIC LabEx, Fondation de Coopération Scientifique Bourgogne-Franche Comté, F-21000 Dijon, France; 4 Valorex, La Messayais, 35210 Combourtillé, France

**Keywords:** palmitic acid, *n*-3 fatty acids, gastrointestinal barrier, intestinal goblet cells, endoplasmic reticulum stress, Muc2 secretion

## Abstract

Diets high in saturated fatty acids (FA) represent a risk factor for the development of obesity and associated metabolic disorders, partly through their impact on the epithelial cell barrier integrity. We hypothesized that unsaturated FA could alleviate saturated FA-induced endoplasmic reticulum (ER) stress occurring in intestinal secretory goblet cells, and consequently the reduced synthesis and secretion of mucins that form the protective mucus barrier. To investigate this hypothesis, we treated well-differentiated human colonic LS174T goblet cells with palmitic acid (PAL)—the most commonly used inducer of lipotoxicity in in vitro systems—or *n*-9, *n*-6, or *n*-3 unsaturated fatty acids alone or in co-treatment with PAL, and measured the impact of such treatments on ER stress and Muc2 production. Our results showed that only eicosapentaenoic (EPA) and docosahexaenoic (DHA) acids protect goblet cells against ER stress-mediated altered Muc2 secretion induced by PAL, whereas neither linolenic acid nor *n*-9 and *n*-6 FA are able to provide such protection. We conclude that EPA and DHA could represent potential therapeutic nutrients against the detrimental lipotoxicity of saturated fatty acids, associated with type 2 diabetes and obesity or inflammatory bowel disease. These in vitro data remain to be explored in vivo in a context of dietary obesity.

## 1. Introduction

Obesity is a complex and multifactorial pathology that is linked to low-grade systemic inflammation, which is identified as a key factor in its development and of related metabolic disorders [1,2]. Systemic inflammation has been shown to be closely related to intestinal microbiota dysbiosis during high-fat (HF) feeding in both mice [3] and humans [4]. A strong increase of gut permeability [5,6] associated with systemic inflammation is partly attributed to the downregulation of genes encoding tight junctions, such as zonula-occludens 1 and occludin [6,7]. A dense mucus layer protects the underlying colonic epithelium against injuries from luminal bacteria and the external environment [8]. The major macromolecular component of this thick barrier is the glycoprotein Muc2, produced by the intestinal goblet cells [9]. Muc2 contains a protein core with cysteine-rich and highly O-glycosylated domains requiring extensive post-translational modifications within the endoplasmic reticulum (ER) and the Golgi body [10].

The high complexity of the Muc2 protein and the strong secretory capacity of goblet cells make Muc2 susceptible to unfolding/misfolding, leading to ER stress [11]. ER stress is emerging as an important contributor to many chronic diseases, such as obesity and obesity-related metabolic disorders, including dyslipidemia and insulin resistance [12,13]. Indeed, studies in both cellular and mouse models [14], as well as obese individuals [15,16], have demonstrated increased fat mass resulting in chronic ER stress in liver and adipose tissues, leading to the development of insulin resistance and type 2 diabetes [17]. Mice fed an HF diet exhibited a significant reduction (−46%) in the intestinal mucus layer, reducing the ability to limit host cells from bacterial infection and resulting in increased colonic and systemic inflammation [18] Mechanistically, this involves activation of the unfolded protein response (UPR), initiating ER stress in epithelial cells and reducing the transcription of proteins such as Muc2, as well as lowering the expression of the goblet cell differentiation factor KLF4 [11]. Similar phenomena have also been observed with ER stressors [19].

Moreover, prolonged ER stress activates the release of free fatty acids from adipocytes, which may contribute, in addition to a high level of saturated fatty acids (FA) brought by obesogenic diets, to lipotoxicity and insulin resistance [20]. Several mouse models, such as *Winnie*, *Eeyore*, and *Kenny*, exhibiting missense mutations in the Muc2 protein, illustrate the link between intestinal cell ER stress and intestinal inflammation [21,22,23]. Prolonged HF diets rich in saturated FA induce colonic epithelial cell ER stress and inflammation [11] and exacerbate mucosal tissue damage in mouse models of spontaneous colitis (Muc2^−/−^, TNF^ΔARE^ mice) [24].

Results over the past decade from both animal and human studies have highlighted the protective effects of *n*-3 polyunsaturated fatty acids (PUFAs) on obesity and associated metabolic disorders. Thanks to their functional properties, they can be considered useful for fighting metabolic syndrome in obese animal models, since they are able to significantly decrease the body weight and fat mass [25,26,27] and improve glucose [28] and lipid metabolism [29]. Dietary *n*-3 PUFAs can notably maintain gut integrity by up-regulating the expression of tight junctions [6,30], improving the epithelial barrier function [31] and lowering permeability-induced inflammatory cytokines [32]. Moreover, alterations in gut microbiota represent an important factor contributing to the beneficial effects of *n*-3 PUFAs in reducing endotoxemia [6]. Importantly, fatty acids have differing effects on ER stress and subsequent inflammatory processes, for example, saturated FA (such as palmitic acid) induce ER stress [33,34,35], while *n*-3 PUFAs (such as docosahexaenoic acid) significantly decrease ER stress in different cellular models [36,37,38].

While numerous studies have reported the extensive beneficial effects of *n*-3 PUFAs in different conditions, to the best of our knowledge, the effects of *n*-3 PUFAs on palmitic acid-induced ER stress in relation to mucin production remain unknown. Therefore, using the well-differentiated human intestinal goblet LS174T cells secreting the mucin Muc2, the aim of the present study was to explore the effect of different unsaturated FAs (exhibiting various chain lengths and unsaturation degrees) to counteract palmitic acid-induced ER stress in colonic epithelial cells. Our data indicate that palmitic acid-altered Muc2 production is alleviated by eicosapentaenoic acid (EPA) and docosahexaenoic acid (DHA) in LS174T cells and that such regulation is partly mediated through ER stress downregulation.

## 2. Materials and Methods

### 2.1. Reagents

Modified Eagle’s medium (MEM), trypsin, fetal bovine serum (FBS), glutamine, and antibiotics were purchased from Dutscher Laboratories. All purified FA (palmitic acid, (PAL), DHA, EPA, α-linolenic acid (LNA), oleic acid (OA), arachidonic acid (AA), and linoleic acid (LA)) and FA-free bovine serum albumin (BSA), thapsigargin, and 4-phenyl butyrate acid (PBA) were purchased from Sigma Aldrich (Saint Quentin Fallavier, France). The antibodies raised against CCAAT-enhancer-binding protein homologous protein (CHOP) and activating transcription factor 4 (ATF4) were purchased from Cell Signaling Technology (Ozyme, France). The horseradish peroxidase (HRP)-linked secondary antibodies were obtained from Jackson ImmunoResearch Laboratories (Interchim, France). The antibody raised against β-actin (C4) HRP was purchased from Santa Cruz. The Muc2 Elisa Kit (Human) was purchased from Aviva Systems Biology (Clinisciences, Nanterre, France).

### 2.2. Cell Culture and Treatments

The LS174T, a well-differentiated human colonic goblet cell line [19], was purchased from Sigma-Aldrich (product number 87060401-1VL Saint Quentin Fallavier, France). Cells were routinely grown as a monolayer in 75 cm^2^ plastic flasks and cultured at 37 °C in a humid environment containing 5% CO_2_ in MEM medium supplemented with 2 mM L-Glutamine (Dutscher, Brumath, France); 10% heat-inactivated fetal bovine serum (FBS); 1% non-essential amino acids (Dutsche, Brumath, France); and an antibiotics cocktail containing 100 U/mL penicillin, and 100 mg/mL streptomycin and amphotericin B (Dutscher, Brumath, France). Cells were seeded in six-well plates with 4 × 10^5^ cells/well. When cells reached 80% confluence, they were treated with different stimuli. PAL, DHA, EPA, LNA, OA, AA, and LA were dissolved in 100% ethanol at a 1 mM concentration, flushed with nitrogen, and stored at −20 °C until use. Before treatment, fatty acids were placed at room temperature, except for PAL, which was heated in a 40 °C water bath. Fatty acids were added to 1 mL of MEM containing FA-free BSA to be combined at a 4:1 ratio. The specific treatment concentrations and incubation times are shown in the figure legends. For the control, cells were treated with the same quantity of ethanol and MEM-BSA concentrations as cells treated with FA.

### 2.3. Western Blot Analysis

LS174T cells were washed three times with ice-cold PBS and cell lysates were obtained in RIPA protein lysis buffer containing 50 mM Tris pH 8.0, 150 mM NaCl, 1% NP (4-Nonylphenyl Poly(ethylene glycol)-40, 0.1% SDS, and 0.5% sodium deoxycholate, and a cocktail of protease and phosphatase inhibitors (Sigma-Aldrich, Saint Quentin Fallavier, France). The cells were kept for 15 min at 4 °C and disrupted by repeated aspiration through a 21-gauge needle. Cell lysates were sonicated and centrifuged at 15,000 g for 15 min at 4 °C and supernatants containing proteins were collected. The protein concentration was determined by a Bicinchoninic acid (BCA) assay. Thirty micrograms of total proteins were subjected to 12% polyacrylamide gel electrophoresis (SDS-PAGE) and electroblotted to a Protan nitrocellulose membrane (Whatman, Dassel, Germany). After blocking non-specific binding sites with 5% free protease BSA in Tween-Tris-buffered saline (T-TBS, 0.1% Tween-20 in TBS) for 1 h, membranes were probed overnight at 4 °C under gentle agitation with primary CHOP and ATF4 antibodies (Cell Signaling, Ozyme, Saint-Cyr-l’École, France) and β-actin (C4) HRP (Santa Cruz Biotechnology, Heidelberg, Germany) at a concentration of 1/1000 and 1/5000, respectively. Blots were then washed three times in T-TBS for 10 min each, and incubated for 1 h at room temperature with a horseradish peroxidase-conjugated secondary antibody at a concentration of 1/5000 for all the antibodies, except β-actin (C4), which was HRP conjugated. The detection of proteins was performed using the enhanced chemiluminescence (ECL) western blotting analysis procedure (Clarity Western ECL, Biorad, Marnes-la-Coquette, France), and their intensity was analyzed with the Chemidoc Imaging System (Biorad, Marnes-la-Coquette, France).

### 2.4. Muc2 ELISA Quantification

After the different treatments, the supernatant was centrifuged at 1000 g for 20 min at 4 °C to remove cells debris and 100 µL per sample was immediately used. The Muc2 Elisa Kit, purchased from Aviva Systems Biology (MUC2 ELISA Kit human, #OKEH02839, Clinisciences, Nanterre, France), was used to perform Muc2 quantification in the supernatant of LS174T cell cultures, following the manufacturer’s instructions.

### 2.5. Total RNA Extraction and Real-Time Quantitative PCR

Total RNA extraction and quantitative RT-PCRs (QRT-PCRs) were performed as previously described [6]. Briefly, LS174T cells were washed three times with ice-cold PBS and homogenized in Tri-Reagent (Euromedex, Souffelweyersheim, France). Total RNA (1 µg) was next reverse transcribed using the High-Capacity RNA-to-cDNA^TM^ kit (Applied Biosystems, France). Q-PCRs were performed using the StepOne Plus real-time PCR system (Applied Biosystems, France) and were carried out using the following human specific primers synthesized by Eurogentec Company (Angers, France): Muc2 (F-5’CAG CAC CGA TTG CTG AGT TG3’, R-5’GCT GGT CAT CTC AAT GGC AG3’), KLF4 (F-5’AGA GGA GCC CAA GCC AAA GA3’, R-5’CAG TCA CAG TGG TAA GGT TTC TC3’), glucose-related protein 78 kDa (GRP78) (F-5’TGC TGC TAG GCC TGC TCC GA3’, R-5’CGA CCA CCG TGC CCA CAT CC3’), CHOP (F-5’CTG CCT TTC ACC TTG GAG AC3’, R-5’ CGT TTC CTG GGG ATG AGA TA 3’), ATF4 (F-5’ATG GCC GGC TAT GGA TGA T3’, R-5’CGA AGT CAA ACT CTT TCA GAT CCA TT3’), and β-actin (F-5’ATG ATA TCG CCG GGC TCG TCG TC3’, R-5’AGG TCC CGG CCA GCC AGG TCCAG3’). Finally, threshold cycle values were calculated by using Step One Software version 2.3 (Life technologies, Illkirch, France). Expression levels of target genes were normalized with the housekeeping gene β-actin and the 2^−ΔΔCt^ method was used to compare the relative expression of gene expression.

### 2.6. Statistical Analyses 

One-way ANOVA was used to determine significance between different conditions. Tukey’s multiple comparison test was used as a post-hoc comparison. For stimulation time-dependent gene expression data, one-way ANOVA with a Tukey’s multiple comparison test as a post-hoc comparison was used to determine the significance difference of treatment at individual time points. The data collected for the two groups were analyzed by an unpaired two-tailed student T-test (* *p* < 0.05; ** *p* < 0.01; *** *p* < 0.001). GraphPad Prism version 7.00 (GraphPad Software Inc., San Diego, CA, USA) was used for the statistical analysis.

## 3. Results

### 3.1. Effects of Palmitic Acid on Muc2 and KLF4 Expression and Muc2 Production in LS174T Cells

**Figure 1:**
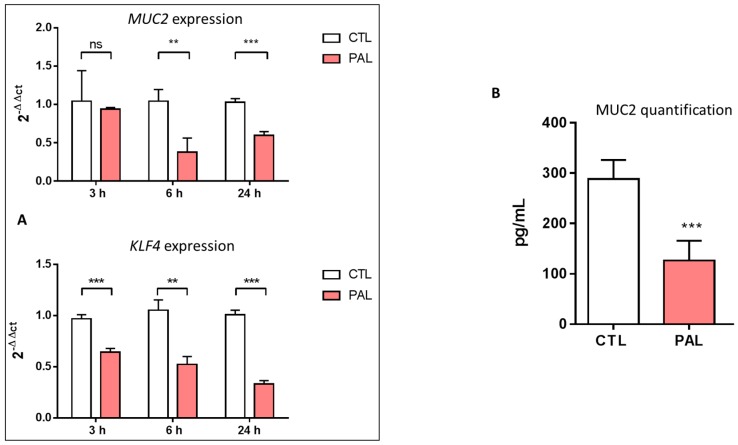
Effects of palmitic acid on *MUC2* and *KLF4* expressions and MUC2 production in LS174T cells. LS174T cells were incubated for 3, 6, or 24 h with PAL (300 µM), or BSA as the control. Cells were harvested, the total mRNA was isolated, and the levels of Muc2 and KLF4 (**A**) expressions were analyzed by real-time quantitative PCR. (**B**): After 24 h of treatment of LS174T cells with PAL, Muc2 quantification was performed in cell culture supernatants by ELISA. Data are expressed as the mean ± SEM of three independent experiments. ** *p* < 0.01 and *** *p* < 0.001 versus CTL-treated groups (Student *t* test). ns: non-significant; CTL: control bovine serum albumin (BSA); PAL: palmitic acid.

Since an HF diet (rich in PAL) was reported to induce a strong colon mucus layer thickness decrease in mice [11,39], we first wanted to assess the effects of PAL on Muc2 and KLF4 mRNA expressions and its impact on Muc2 secretion, the main mucin secreted by the LS174T cell line. We observed that 300 µM of PAL (considered a mildly elevated concentration) downregulated Muc2 mRNA expression after 6 and 24 h of treatment. Moreover, a significant decrease in goblet cell differentiation (KLF4) was observed after 3, 6, and 24 h of treatment with PAL (Figure 1A). Furthermore, to determine whether PAL affects secretion of the Muc2 protein by LS174T cells, Muc2 quantification was performed in the culture medium after 24 h of treatment. The level of the mucous glycoprotein Muc2 was clearly reduced in the culture medium of LS174T cells treated with PAL for 24 h (Figure 1B). These different data showed that PAL altered not only Muc2 and KLF4 expressions, but also Muc2 release by the LS174T cells, with no change in their viability at this concentration (300 µM) of PAL (Figure S1).

### 3.2. Effects of Palmitic Acid on Endoplasmic Reticulum Stress in LS14T Cells

**Figure 2:**
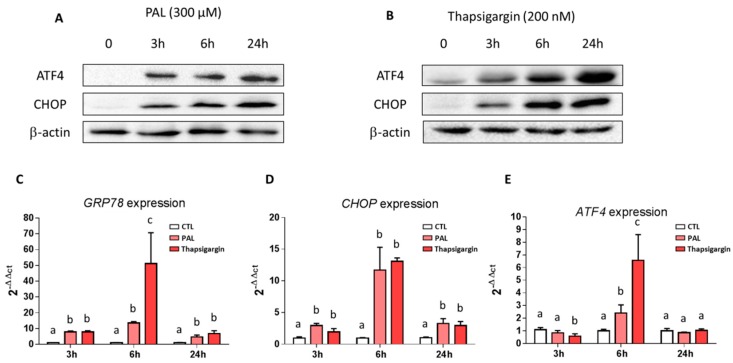
Effects of palmitic acid on endoplasmic reticulum stress in LS174T cells. Cells were treated for 3, 6, or 24 h with PAL (300 µM) or thapsigargin (200 nM) as a strong inducer of ER stress. BSA was used as the control. Representative western blots for ATF4 and CHOP are shown for PAL (**A**) and thapsigargin (**B**) treatments. mRNA expressions of UPR genes GRP78 (**C**), CHOP (**D**), and ATF4 (**E**) in LS174T were measured by quantitative RT-PCR, expressed relatively to CTL and normalized to actin. Data are represented as the mean ± SEM of three independent experiments. Differences were analyzed by Tukey’s multiple comparison test. Bars assigned different superscript letters (a, b, c) were statistically different at *p* < 0.05. ns: non-significant; ATF4: activating transcription factor 4; CHOP: CCAAT-enhancer-binding protein homologous protein; CTL: control bovine serum albumin (BSA); ER: endoplasmic reticulum; GRP78: glucose-related protein 78 kDa; PAL: palmitic acid.

The high secretory output of the goblet cell makes Muc2, containing highly glycosylated domains, prone to misfolding, and failure to resolve this misfolding leads to ER stress, as shown in high-fat diets [11]. With this is mind, we investigated the impact of PAL on ER stress, by treating LS174T cells with PAL at various times ranging from 3 to 24 h and next evaluated markers of ER stress. ATF4 (activating transcription factor 4) and CHOP (CCAAT-enhancer-binding protein homologous protein) protein expressions were drastically increased in PAL-treated LS174T cells, whatever the duration of the treatment (Figure 2A). Comparable results were observed by the use of thapsigargin as a positive control (Figure 2B). Moreover, whatever the time of treatment, a clear increase in the mRNA expression of ER stress markers, GRP78 (glucose-related protein 78 kDa), and CHOP was observed in LS174T cells treated with PAL (Figure 2C,D). ATF4 gene expression was increased at 6 h after PAL treatment, whereas it remained unchanged at 3 and 24 h (Figure 2E).

### 3.3. Preventing Endoplasmic Reticulum Stress Restores Palmitic Acid-Altered Muc2 Secretion

**Figure 3:**
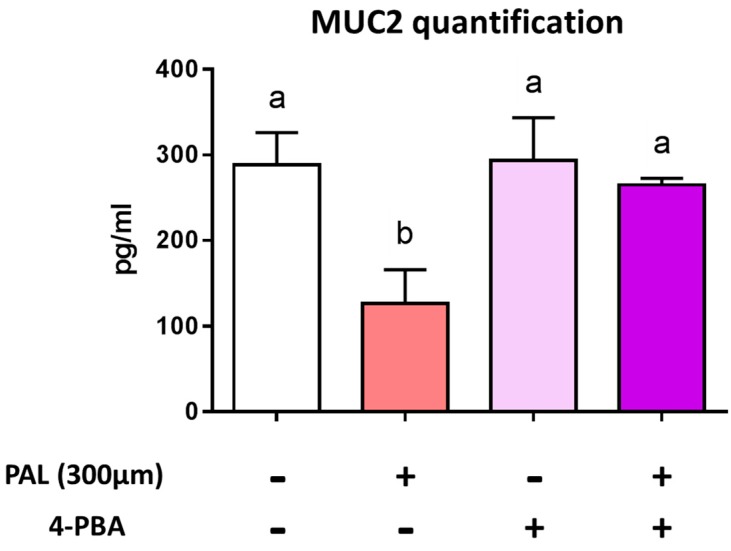
Preventing endoplasmic reticulum stress restores palmitic acid-altered MUC2 secretion. LS174T cells were treated for 24 h with control BSA, 300 µM PAL, or 500 µM 4-PBA, or were co-treated with PAL and 4-PBA. After incubation, cell culture supernatants were centrifuged to remove cell debris and the Muc2 protein released by LS174T cells was then quantified by ELISA. Results are presented as the mean ± SEM. of three independent experiments. Differences were analyzed by Tukey’s multiple comparison test. Bars assigned different superscript letters (a, b, c) were statistically different at *p* < 0.05. BSA: bovine serum albumin; PAL: palmitic acid; 4-PBA: 4-phenyl butyrate acid.

We next co-treated LS174T cells with PAL and 4-phenylbutyrate (4-PBA), an ER stress inhibitor, in order to verify whether altered Muc2 secretion is due to ER stress induced by PAL. After 24 h of treatment with PAL, the Muc2 concentration significantly decreased in the culture medium of LS174T cells, whereas co-treatment with 4-PBA normalized Muc2 secretion (Figure 3). These results evidenced that the altered Muc2 release observed in PAL-treated LS174T cells mainly occurs through the triggering of ER stress.

### 3.4. Only EPA and DHA Prevent Endoplasmic Reticulum Stress Induced by Palmitic Acid

**Figure 4:**
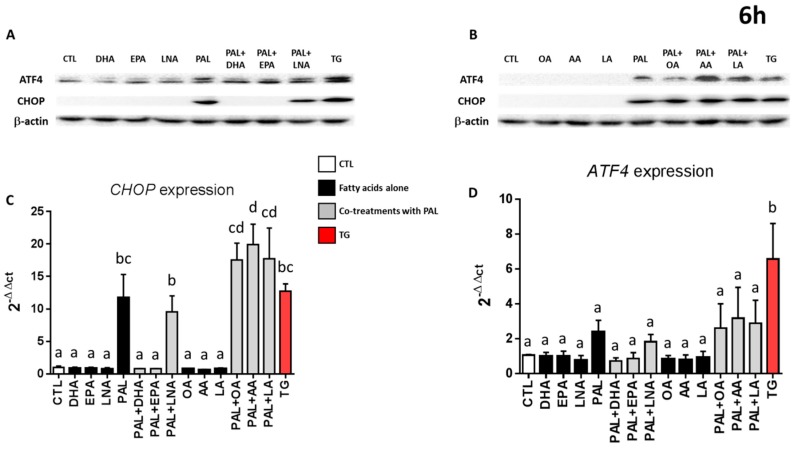
Only EPA and DHA prevent endoplasmic reticulum stress induced by palmitic acid. LS174T cells were treated for 6 h with control BSA, 300 µM PAL, or 200 nM thapsigargin alone, or with 25 µM DHA (docosahexaenoic acid), EPA (eicosapentaenoic acid), LNA (linolenic acid), OA (oleic acid), AA (arachidonic acid), or LA (linoleic acid) alone or in co-treatment with 300 µM PAL. After 6 h of treatment, cell lysates were immunoblotted for ATF4, CHOP, and actin. Representative western blots are presented in (**A**) and (**B**). The impact of 6 h of treatment with the different fatty acids and the ER stress inducer thapsigargin on the mRNA expressions of CHOP and ATF4 were analyzed by quantitative RT-PCR. Results are expressed relatively to CTL and normalized to β-actin (**C**) and (**D**). Data are expressed as the mean ± SEM of three independent experiments. Differences were analyzed by Tukey’s multiple comparison test. Bars assigned different superscript letters (a, b, c) were statistically different at *p* < 0.05. CTL: control bovine serum albumin (BSA); TG, thapsigargin; ATF4: activating transcription factor 4; CHOP: CCAAT-enhancer-binding protein homologous protein; ER: endoplasmic reticulum.

As *n*-3 fatty acids have been shown to prevent ER stress in both in vitro [36,40] and in vivo models [41,42,43], we investigated the interaction between PAL, *n*-3, *n*-6, and monounsaturated FA upon ER stress. As observed above (Figure 2), the protein expression levels of CHOP and ATF4 were increased when LS174T cells were treated with PAL for 6 h. Moreover, co-treatment with PAL and -linolenic acid, linoleic acid, arachidonic acid, or oleic acid (Figure 4A,B) also overexpressed ATF4 and CHOP protein expression. More interestingly, the upregulation observed with PAL was lowered only when PAL was combined with 25 µM of DHA or EPA (Figure 4A). We next evaluated CHOP and ATF4 mRNA expressions in the same conditions. PAL overexpressed CHOP and similarly tended to increase ATF4, as already observed in Figure 2. Co-treatment of PAL with EPA and DHA alleviated CHOP and tended to reduce ATF4 overexpression, whereas LNA, OA, AA, and LA did not (Figure 4C,D). Similar results were obtained after 3 and 24 h of treatment (Figure S2).

### 3.5. EPA and DHA Prevent Palmitic Acid-Altered Muc2 and KLF4 Expressions and Muc2 Production in LS174T Cells

**Figure 5:**
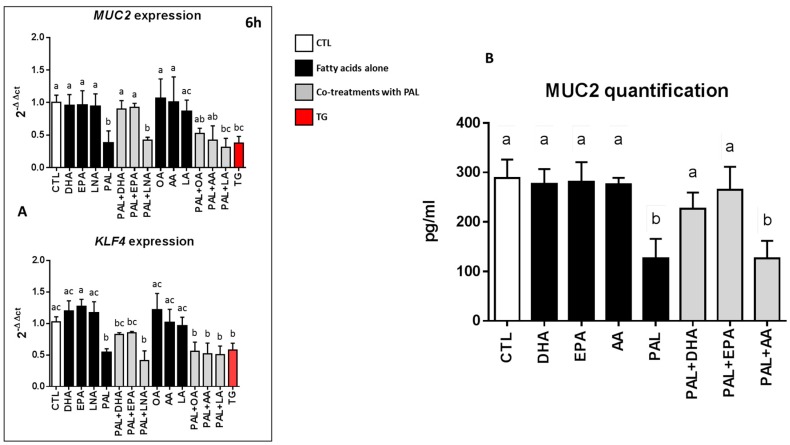
EPA and DHA prevent palmitic acid-altered *MUC2* and *KLF4* expressions and MUC production in LS174T cells. LS174T cells were treated for 6 h with control BSA, 300 µM PAL, or 200 nM thapsigargin alone, or with 25 µM DHA, EPA, LNA, OA, AA, or LA alone or in co-treatment with 300 µM PAL. (**A**): The mRNA expressions of Muc2 and KLF4 were evaluated by quantitative RT-PCR after 6 h of treatment with the different fatty acids and the ER stress inducer thapsigargin. The mRNA expressions are relative to CTL and normalized to actin. (**B**): After treatment for 24 h with the different fatty acids, cell culture supernatants were centrifuged to remove cell debris and the Muc2 protein produced by LS174T cells was then quantified by ELISA. Data are expressed as the mean ± SEM of three independent experiments. Differences were analyzed by Tukey’s multiple comparison test. Bars assigned different superscript letters (a, b, c) were statistically different at *p* < 0.05. CTL: control bovine serum albumin (BSA); ER: endoplasmic reticulum.

As observed above (Figure 1A), Muc2 and KLF4 mRNA expressions were downregulated when LS174T cells were treated with PAL for 6 h. Co-treatments with PAL and LNA, LA, AA, or OA also significantly decreased Muc2 and KLF4 mRNA expressions. Nevertheless, when EPA and DHA were added to PAL-treated LS174T cells, Muc2 and KLF4 mRNA expressions remained comparable to those of the control (Figure 5A). Similar results were obtained at 24 h of treatment (Figure S3). Lastly, when the level of the mucous glycoprotein Muc2 in the culture medium of LS174T cells was not affected by treatment with EPA, DHA, or AA alone, it was statistically reduced with PAL and with PAL and AA. In contrast, the secretion of Muc2 remained similar to the control when EPA and DHA were added to PAL (Figure 5B).

## 4. Discussion

Obesity is now widely known to be associated with low-grade systemic inflammation. Indeed, it has been shown that HF diets, rich in saturated FA and particularly PAL, strongly increase intestinal permeability, leading to lipopolysaccharide absorption and metabolic endotoxemia that triggers inflammation and metabolic disorders [1,2,44]. The mucus layer, mainly comprised of the glycoprotein Muc2 produced by intestinal goblet cells [45], forming a physical barrier protecting the underlying epithelium against luminal substances and microbes [46,47], has been shown to be considerably altered under diets rich in saturated FA [11,18], exacerbating epithelium leakage and endotoxemia. We and others have evidenced that mice enriched in *n*-3 polyunsaturated fatty acids (PUFAs) are protected against gut barrier dysfunction, with consequences on metabolic endotoxemia [6,48,49,50]. However, the effects of *n*-3 PUFAs on the secretory function of intestinal goblet cells remain largely unexplored. Using the well-differentiated human colonic goblet LS174T cell line, the present study shows that PAL decreases Muc2 production, mainly by generating a rise of ER stress in LS174T cells which detrimentally affects the production of the secreted mucosal barrier. More interestingly, we evidenced here, for the first time, that long-chain *n*-3 FA (C20 and C22 carbon chain length) are able to prevent the altered Muc2 production induced by PAL, mainly by alleviating ER stress.

Our results agree with previous reports showing that PAL induces ER stress in many cell types [33,34,51,52] and particularly with the one by Gulhane and co-workers, who showed that 500 µM of PAL induced significant stress in LS174T cells with a decrease in Muc2 and the goblet cell differentiation transcription factor KLF4 mRNA expressions [11]. These mRNA downregulations were also accompanied in the present study by a significant decrease of the secretion of the Muc2 protein by the cells (Figure 1). This result can be explained by the fact that PAL reduces the production of mature fully glycosylated Muc2 accompanied by an increase of the non-glycosylated Muc2 precursor and a reduction in mature Muc2 secretion, demonstrating protein misfolding consistent with the unfolded protein response (UPR) activation observed [11]. Due to their large size and complexity, mucins are extremely susceptible to misfolding in the ER, which can eventually lead to ER stress. We presently observed that PAL strongly increased ER stress in LS174T cells (Figure 2), which has also been observed with other ER stressors, such as thapsigargin, in the present study or tunicamycin in others [19]. In vivo, a decreased Muc2 secretion resulted in a thinner mucus layer more easily penetrated by diffusing microbial products, and more easily degraded by mucin-degrading bacteria. This is supported by previous studies evidencing that HF diet-induced obese mice exhibit a 50% thinner colon mucus layer [18]. Then, we may assume that the altered thickness of the mucus layer by an obesogenic diet (rich in PAL) could be explained by mucin misfolding leading to ER stress and an UPR-activated inhibition of Muc2 transcription with a downstream effect of reducing Muc2 production. This assumption is strengthened here by the use of the chemical ER chaperone 4-PBA (an ER stress inhibitor), which was able to prevent PAL-induced alteration of Muc2 production by LS174T cells (Figure 3).

To our knowledge, the present study is the first to demonstrate that ER stress produced by PAL in goblet LS174T cells can be significantly reduced by EPA and DHA, two highly unsaturated FA of the *n*-3 series exhibiting strong anti-inflammatory properties [53]. This is mechanistically supported by a decrease in the raised levels of UPR-related gene expression of CHOP and ATF4 associated with PAL in LS174T cells (Figure 4). In vitro studies have already suggested that DHA is able to counteract palmitate-induced ER stress in different cell types, including primary mouse hepatocytes [37], mouse 3T3L1 and rat primary preadipocytes [54], pancreatic cells [36,38], and C2C12 myotubes [40], but it has never been studied in colon cells in relation to mucus secretion. We evidenced in the present study that EPA and DHA are able to prevent altered Muc2 production in PAL-treated LS174T cells (Figure 5B). This protective effect of EPA and DHA can be explained by ER stress alleviation, as shown Figure 4. The well-known anti-inflammatory effects of EPA and DHA can also be involved in the modulation of altered goblet cell homeostasis and the decreased production of mucin. Indeed, resolvins—derived from EPA and DHA—due to their anti-inflammatory properties, have also been suggested to attenuate ER stress-induced apoptosis in HepG2 cells, mainly through the JNK pathway [55]. Moreover, it has also been suggested that *n*-3 PUFA suppression of ER stress was partly due to AMP-activated protein kinase (AMPK) activation. In support of this, compound C (an AMPK inhibitor) is able to block the effects of DHA in PAL-induced ER stress inhibition [41].

LNA was supposed to have a similar efficiency to EPA and DHA against PAL lipotoxicity via reducing ER stress and apoptosis, as already shown in primary rat hepatocytes [56] or renal NRK-52E cells [57]. Unexpectedly, while LNA is also a *n*-3 fatty acid, it failed to prevent PAL alterations, as observed with EPA or DHA. Indeed, the expression of CHOP, ATF4, and Muc2 in LS174T cells co-treated with PAL and LNA remained similar to the one observed with PAL alone, whereas it was changed at least twice when cells were co-treated with PAL and EPA or DHA (Figure 4 and Figure 5). Then, EPA, DHA, and LNA would be able to differently modulate PAL metabolism and consequently its lipotoxic effect. This has already been observed in other cell types, such as C2C12 myoblasts [58] and L6 myotubes [59], where partial and total oxidation were decreased during PAL treatment, which was restored by EPA and DHA, but not with LNA [58,59]. Then, we might expect comparable modulations in the present study. Moreover, besides alterations in its oxidation, it has also been shown that PAL treatment increased the formation of lipotoxic compounds, such as diglycerides and ceramides [60], and that EPA or DHA were able to 1) reduce such accumulation, 2) enhance triglyceride synthesis, and 3) preferentially address PAL to mitochondrial oxidation [58,61]. In contrast and in agreement with Pinel and coworkers [58], LNA failed to prevent PAL incorporation into cytotoxic diglycerides, and then to reduce the related ER stress activation and finally Muc2 production. Therefore, our results highly suggest that LNA intake in a diet high in *n*-3 fatty acids would not represent an alternative to oily fish consumption (rich in EPA and DHA) with regard to gut barrier integrity and especially to the preservation of the thickness of the mucus layer. In addition, a greater expression (+75%) of CHOP was observed in cells co-treated with PAL and AA compared with PAL alone (Figure 4). This increase could be due to the proinflammatory effects of AA-derived prostanoids and leukotrienes, as previously described [62], and supports the idea that certain *n*-6 fatty acids may have detrimental effects when consumed excessively.

In short, our results suggest that very long-chain *n*-3 PUFAs, by protecting goblet cells against ER stress-mediated altered Muc2 secretion induced by PAL, could be potential therapeutic nutrients used in strategies against the detrimental lipotoxicity of saturated FA, associated with type 2 diabetes and obesity or inflammatory bowel disease. Nevertheless, the relevance of our in vitro data remain to be explored in vivo in a context of dietary obesity, as well as the efficiency of *n*-3 PUFAs in alleviating the decrease of the thickness of the intestinal mucus layer and consequently preserving gut barrier integrity.

## Supplementary Materials

The following are available online at https://www.mdpi.com/2072-6643/11/9/2179/s1: Figure S1: Effect of palmitic acid and thapsigargin treatments on the viability of LS174T cells; Figure S2: EPA and DHA prevent endoplasmic reticulum stress induced by palmitic acid at 3 and 24 h; Figure S3: EPA and DHA prevent palmitic acid-altered Muc2 and KLF4 expressions in LS174T cells at 3 and 24 h.
