# Supplementary material for: Docosahexaenoic and Eicosapentaenoic Acids Prevent Altered-Muc2 Secretion Induced by Palmitic Acid by Alleviating Endoplasmic Reticulum Stress in LS174T Goblet Cells
Source: Nutrients. 2019 Sep 11;11(9):2179. doi: 10.3390/nu11092179 (PMC6770956; doi:10.3390/nu11092179)
Supplement: Supplementary file 1 [file nutrients-11-02179-s001.pdf]

Figure S1 : Effect of palmitic acid and thapsigargin treatments on viability of LS174T cells.

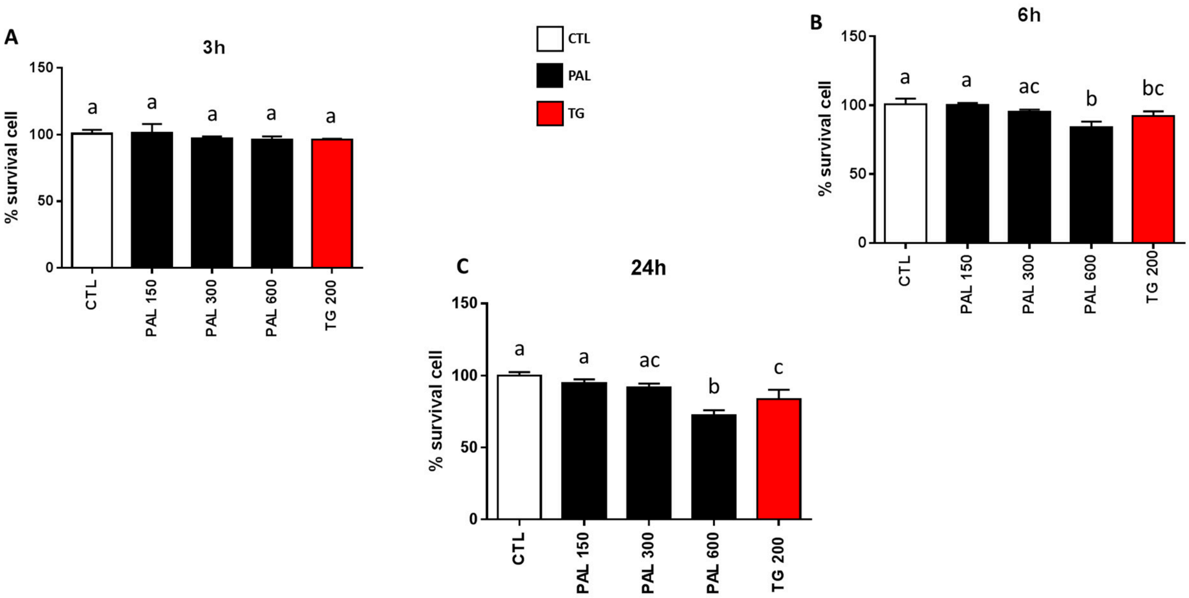

Figure S1. Effect of palmitic acid and thapsigargin treatments on viability of LS174T cells. Cell viability measured after exposure to various concentrations (150, 300 or 600 μM) of PAL or 200 nM of thapsigargin for 3 h (**A**), 6 h (**B**) or 24 h (**C**) by the use of crystal violet dye solution. Data are represented as mean ± SEM of three independent experiments. Differences were analyzed by Tukey's multiple comparison test. Bars assigned different superscript letters (a,b,c) were statistically different at $p < 0.05$. CTL: control BSA; PAL: palmitic acid; TG: thapsigargin.

Figure S2 : EPA and DHA prevent endoplasmic reticulum stress induced by palmitic acid at 3 and 24 hours

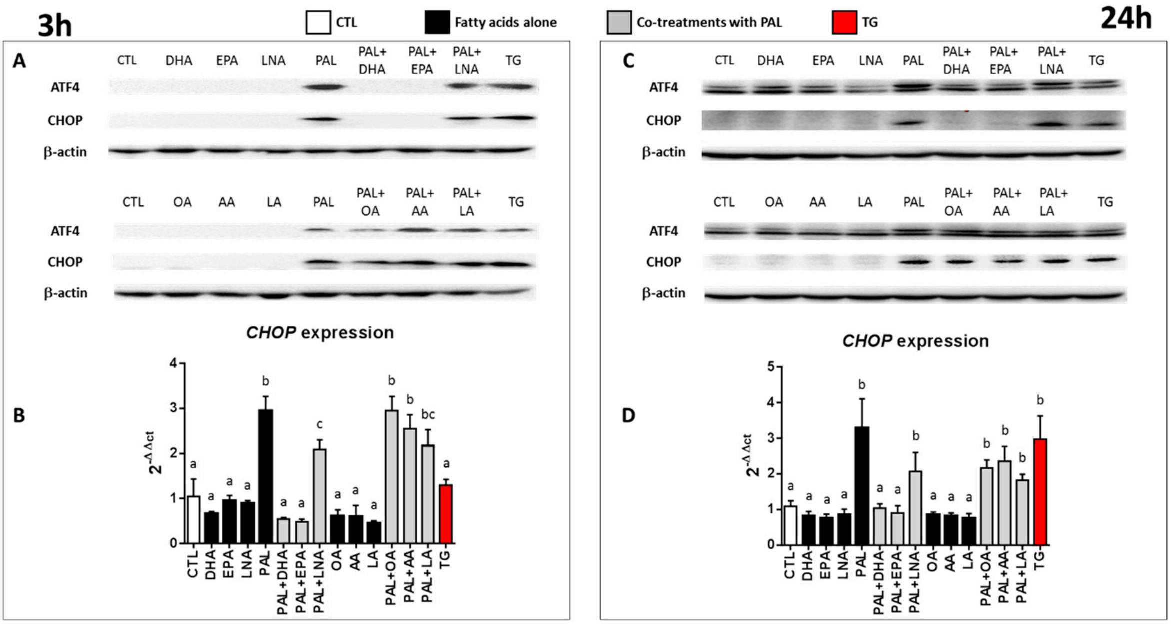

Figure S2. EPA and DHA prevent endoplasmic reticulum stress induced by palmitic acid at 3 and 24 hours. LS174T cells were treated for 3 h or 24 h with control BSA or with 300 μM PAL or 200 nM thapsigargin alone, or with 25 μM DHA, or EPA, or LNA, or OA, or AA or LA alone or in co-treatment with 300 μM PAL. After 3 or 24 h of treatment, cell lysates were immunoblotted for ATF4, CHOP and β-actin. Representative western blots are presented in (**A**) and (**C**). The impact of 3 or 24 h of treatment with the different fatty acids and the ER stress inducer thapsigargin on the mRNA expressions of *CHOP* was analyzed by quantitative RT-PCR. Results are expressed relatively to CTL and normalized to β-actin (**B**) and (**D**). Data are expressed as mean ± SEM of three independent experiments. Differences were analyzed by Tukey's multiple comparison test. Bars assigned different superscript letters (a,b,c) were statistically different at $p < 0.05$. CTL: control BSA; DHA: docosahexaenoic acid; EPA: eicosapentaenoic acid; LNA: α-linolenic acid; PAL: palmitic acid; OA: oleic acid; LA: linoleic acid; AA: arachidonic acid and TG, thapsigargin.

Figure S3 : EPA and DHA prevent palmitic acid-altered *Muc2* and *KLF4* expressions in LS174T cells at 3 and 24 hours

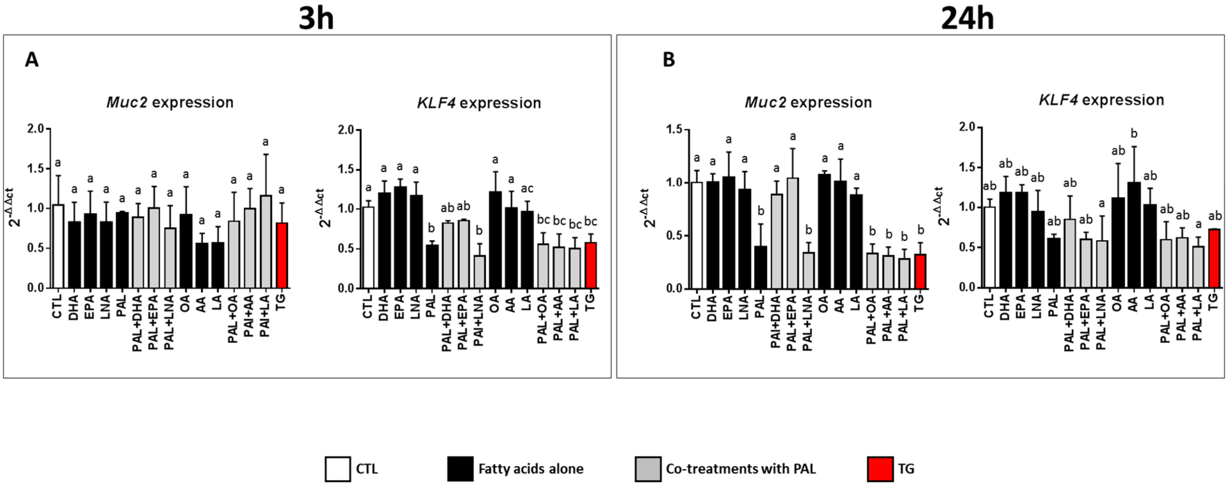

Figure S3. EPA and DHA prevent palmitic acid-altered Muc2 and KLF4 expressions in LS174T cells at 3 and 24 hours. LS174T cells were treated for 3 and 24 h with control BSA or with 300 μM PAL or 200 nM thapsigargin alone, or with 25 μM DHA, or EPA, or NA, or OA, or AA or LA alone or in co-treatment with 300 μM PAL. (**A** and **B**): The mRNA expressions of Muc2 and KLF4 were evaluated by quantitative RT-PCR after 3 and 24 h of treatment with the different fatty acids and the ER stress inducer thapsigargin. The mRNA expressions are relative to CTL and normalized to β-actin. Data are expressed as mean ± SEM of three independent experiments. Differences were analyzed by Tukey's multiple comparison test. Bars assigned different superscript letters (a,b,c) were statistically different at $p < 0.05$. CTL: control BSA; DHA: docosahexaenoic acid; EPA: eicosapentaenoic acid; LNA: α-linolenic acid; PAL: palmitic acid; OA: oleic acid; LA: linoleic acid; AA: arachidonic acid and TG, thapsigargin.
